# Moderate Thermal Stress Causes Active and Immediate Expulsion of Photosynthetically Damaged Zooxanthellae (*Symbiodinium*) from Corals

**DOI:** 10.1371/journal.pone.0114321

**Published:** 2014-12-10

**Authors:** Lisa Fujise, Hiroshi Yamashita, Go Suzuki, Kengo Sasaki, Lawrence M. Liao, Kazuhiko Koike

**Affiliations:** 1 Graduate School of Biosphere Science, Hiroshima University, Higashi-Hiroshima, Hiroshima, Japan; 2 Research Center for Subtropical Fisheries, Seikai National Fisheries Research Institute, Fisheries Research Agency, Ishigaki, Okinawa, Japan; 3 Western Region Industrial Research Center, Higashi-Hiroshima, Hiroshima, Japan; National University of Singapore, Singapore

## Abstract

The foundation of coral reef biology is the symbiosis between corals and zooxanthellae (dinoflagellate genus *Symbiodinium*). Recently, coral bleaching, which often results in mass mortality of corals and the collapse of coral reef ecosystems, has become an important issue around the world as coral reefs decrease in number year after year. To understand the mechanisms underlying coral bleaching, we maintained two species of scleractinian corals (Acroporidae) in aquaria under non-thermal stress (27°C) and moderate thermal stress conditions (30°C), and we compared the numbers and conditions of the expelled *Symbiodinium* from these corals. Under non-thermal stress conditions corals actively expel a degraded form of *Symbiodinium*, which are thought to be digested by their host coral. This response was also observed at 30°C. However, while the expulsion rates of *Symbiodinium* cells remained constant, the proportion of degraded cells significantly increased at 30°C. This result indicates that corals more actively digest and expel damaged *Symbiodinium* under thermal stress conditions, likely as a mechanism for coping with environmental change. However, the increase in digested *Symbiodinium* expulsion under thermal stress may not fully keep up with accumulation of the damaged cells. There are more photosynthetically damaged *Symbiodinium* upon prolonged exposure to thermal stress, and corals release them without digestion to prevent their accumulation. This response may be an adaptive strategy to moderate stress to ensure survival, but the accumulation of damaged *Symbiodinium*, which causes subsequent coral deterioration, may occur when the response cannot cope with the magnitude or duration of environmental stress, and this might be a possible mechanism underlying coral bleaching during prolonged moderate thermal stress.

## Introduction

Coral reefs are habitats that support very high biodiversity in which approximately one-quarter to one-third of all marine species live, despite coral reefs covering only 0.2% of the ocean’s surface [1]–[3]. Reefs mainly consist of scleractinian corals living in symbiosis with zooxanthellae, particularly members of the dinoflagellate genus *Symbiodinium*. *Symbiodinium* are unicellular microalgae that reside within the endodermal tissues of many marine animals and receive inorganic nutrients from them. In return, these symbionts provide photosynthetic products to their hosts [4], [5]. It is estimated that 60–85% of the total nutrition of the hosts is derived from the symbionts, which enable corals to survive in oligotrophic tropical waters [6].

In recent decades, a phenomenon called coral bleaching has become an important issue resulting in recurring mass mortality of corals around the world [7]. “Coral bleaching” is a phenomenon in which the white color of the coral skeleton becomes apparent due to the loss of *Symbiodinium* and/or the loss of their photosynthetic pigments, often resulting in coral death [7]. Coral reefs have been declining year after year, and it is estimated that almost 19% of the world’s coral reefs have disappeared since 1950 [8]. Because of this decline, there is an urgent need to clarify the mechanism behind coral bleaching to be able to conserve coral reefs.

Many environmental triggers are known to induce coral bleaching, such as elevated seawater temperatures (e.g., [9]–[12]), high light intensity (e.g., [10], [13]), salinity stress (e.g., [14], [15]), cold shock (e.g., [11], [16]), and disease (e.g., [17], [18]). Among them elevated seawater temperature is thought to be one of the most significant factor leading to coral bleaching. From 1997–1998, an enormous coral bleaching episode occurred around the world due to abnormally high seawater temperatures caused by El Niño [19]–[26]. Many researchers have attempted to clarify the mechanism of coral bleaching, especially under conditions of elevated water temperature (e.g., [27]–[31]). Their experiments have demonstrated that elevated seawater temperature is a primary trigger of coral bleaching. However, many of these thermal stress experiments were performed at water temperatures greater than 32°C. Under such harsh thermal stress, a large number of *Symbiodinium* were most likely expelled due to host cell detachment, and the subsequent loss of *Symbiodinium* from coral tissues led to coral bleaching [11], [29], [32]. However, many natural coral bleaching events can occur even under conditions of moderate thermal stress, 1–2°C higher than the average ambient seawater temperature, for prolonged periods of time [23], [33]–[36]. For example, in the summer of 2013, coral bleaching was observed around Okinawa Island because the seawater temperature reached 30°C for nearly one month [37]–[39]. There have been numerous experiments using seawater temperatures higher than 32°C, but those under prolonged, though moderate thermal stress are limited [12], [29], [40].

In this study, expulsion of *Symbiodinium* from corals was investigated after exposing the corals under moderate thermal stress conditions (30°C) and compared with those under initial non-thermal stress conditions (27°C). Considering our previous research [32], we specifically monitored the cellular forms of expelled *Symbiodinium* under light and transmission electron microscopes. The expulsion of the degraded (digested) form is a healthy, normal mechanism in corals that maintains the *Symbiodinium* population, whereas the release of normal forms indicates coral degradation. Moreover, to assess damage of expelled *Symbiodinium* cells from each coral, the maximum quantum yield of photosystem II (PSII) (*Fv/Fm*) of individual *Symbiodinium* cells was measured by means of microscopy type of pulse-amplitude modulation (PAM) fluorometer. With this experiment, we clarified the expulsion mechanism of *Symbiodinium* under different thermal stress conditions, which may provide clues to better understand the process behind coral bleaching.

## Materials and Methods

### Collection of corals and aquarium conditions

In October 2012, colonies of *Acropora selago* (Studer, 1878) and *Acropora muricata* (Linnaeus, 1758) were collected from a 2 m depth inner reef off of Ishigaki Island, Okinawa, Japan (24°36′N, 124°19′E). The sampling of corals was permitted for research use as an exception by the Okinawa Prefectural Government (No. 24–54). These coral species were selected as models for this experiment because of their known susceptibility to elevated seawater temperature. The sizes of colonies ranged from 6–20 cm wide, 6–15 cm long, and 3–12 cm high. Coral colonies were initially kept in running seawater tank for five days to acclimate, and two or three coral colonies were placed in a 12 L aquarium, resulting in a total of six aquaria for each species. Colonies in three aquaria for each coral species were used for the repeated collection of coral branches and the isolation of *Symbiodinium* from their tissue, while undisrupted colonies from the other three aquaria were used for collecting *Symbiodinium* expelled into the water for triplicate experiments. These total 12 aquaria (for two species) were placed in a large water bath to maintain the water temperature. The aquaria were aerated with air-stones and maintained with temperature-regulated flowing seawater (using a thermostatic device, GA7500-ODHT-E, Gunji, Osaka, Japan) filtered by a MEMCOR Ultra-filtration unit (0.2 µm pore size membrane module, JFE Engineering, Tokyo, Japan) at a flow-rate of 1 L min^−1^ for each aquarium. Water temperature of each aquarium was measured daily to monitor the temperature variance. Light was provided with four 500 W metal halide lamps hanging over the entire aquaria at a photon-flux density of 150±9 µmol photons m^−2 ^s^−1^ (mean ± SD among the aquaria; measured by a cosine PAR sensor under the water at the same height of corals) with a 12∶12 h light/dark cycle. The experiment was performed at the Research Center for Subtropical Fisheries, Seikai National Fisheries Research Institute, in Okinawa, Japan.

### Temperature treatments

After a five day period of acclimation, the experiment was started. The aim of this study is to compare the *Symbiodinium* expulsion phenomena between non-thermal stress (27°C) and moderate thermal stress conditions (30°C) in the same coral colony, thus, for the first five days of experiment, the temperature was maintained at 26.9±0.15°C (mean ± SD) (non-thermal stress period), and then it was raised in a stepwise manner (0.5°C increase every 8 h) for the next two days (transitional temperature period) until it reached 30°C, where it was maintained for six days (thermal stress period: maintained at 30.2±0.15°C). The temperature treatment was subjected to all the aquaria simultaneously. The samplings from each aquaria were basically conducted under non-thermal stress conditions at 27°C (26.9±0.15°C), under transition temperature conditions during increasing temperature from 27°C to 30°C, and under moderate thermal stress conditions at 30°C (30.2±0.15°C). In this way we were able to compare expulsion phenomena between the two different temperature treatments as well as the transition period between these two treatments. To account for experimental controls, single colony of each species was placed in a spare aquarium at constant 27°C (26.9±0.15°C), and dark-adapted maximum quantum yields of photosystem II (*Fv/Fm*) of freshly isolated *Symbiodinium* were monitored at nearly the end of the experiment (days 11, 12, and 13) using WATER-PAM (Walz, Effeltrich, Germany).

### Collection of coral branches and expelled *Symbiodinium* from the corals

A branch was snipped off of each coral species in three aquaria designated for collection to count the *Symbiodinium* density *in hospite* for three days at 27°C (days 1, 3, and 5) and 30°C (days 9, 11, and 13). The branches were immediately frozen for subsequent processing. Expelled *Symbiodinium* were collected from the aquarium water every day throughout the 13-day experimental period. According to an existing report [41], corals show a daily rhythmicity of *Symbiodinium* expulsion with a peak at noon. Therefore, from 12∶00 to 14∶00, the supply of seawater to the aquaria was stopped, with expelled *Symbiodinium* expected to accumulate in the aquarium water within this two-hour period. To prevent water temperature increases, seawater flow was maintained in the water bath. Four liters of seawater (including expelled *Symbiodinium*) was collected from each aquarium after mixing well and sieving through a 20 µm mesh to remove large particles and then concentrated to 50 mL using a 1 µm mesh. Microscopy confirmed the absence of *Symbiodinium* in the filtrate.

### Counting the expelled *Symbiodinium* and *Symbiodinium* density *in hospite*

The expelled *Symbiodinium* cells in 1 mL of the concentrated sample were trapped in a polycarbonate filter (Isopore membrane filters, 0.8 µm ATTP, ATTP01300, Millipore, Billerica, MA, USA) by gentle vacuuming. The filter was then mounted onto a glass slide, immersed in a drop of mineral oil to prevent dehydration and oxidation, and covered with a cover slip. Samples were kept in the dark and stored in a freezer (–20°C) until observation. The cells on the filter were counted under transmitted light while simultaneously observing chlorophyll *a* auto-fluorescence under an epifluorescence microscope (BX51, Olympus, Tokyo, Japan) with blue light excitation. Transmitted-light and fluorescence micrographs were taken of 20 randomly selected areas using a microscope-mounted camera (Cool Snap ES, Photometrics, Tucson, AZ, USA) under a 20×objective lens. The number of *Symbiodinium* cells was counted based on the morphologies seen in the micrographs: a normal form in which the size was approximately 10 µm and showed bright auto-fluorescence, and a degraded form approximately half the size of the normal form with weak auto-fluorescence and in a condensed state, based on Fujise et al. (2013) [32]. The number of cells in each observation field was averaged for 20 areas, and the total number of cells was determined for 1 mL of the concentrated sample. The number of expelled *Symbiodinium* cells was determined for each aquarium and converted to expelled cells per hour per coral surface area. The skeletons of coral specimens were kept after the experiment and subjected to surface area measurement using a three-dimensional camera (detailed description below).

*Symbiodinium in hospite* were removed from the thawed coral branches collected on days 1, 3, 5, 9, 11, and 13, using an air brush with filtered seawater (0.2 µm mesh filter) and then quantified to 50 mL. *Symbiodinium* cells were counted using a hemocytometer under a light microscope (CKX 41, Olympus, Tokyo, Japan) three times for each branch, and the average number of cells was converted to *Symbiodinium* density considering the coral surface area (cm^2^).

### Measurement of PSII maximum quantum yield of *Symbiodinium*

The dark-adapted maximum quantum yield of photosystem II (*Fv/Fm*) was measured using a microscopy-type PAM fluorometer (Micro-FluorCam FC 2000, Photon Systems Instruments, Brno, Czech Republic). *Symbiodinium* freshly isolated from the host tissue using a water pick and expelled *Symbiodinium* in the concentrated sample were transferred to glass slides, and *Fv/Fm* of the individual cells were measured under the microscopy PAM fluorometer with the following settings: flash (measuring beam) intensity = 20, super (saturation flash) intensity = 60, shutter speed = 100, and CCD sensitivity = 60, with a 20×objective lens. More than 60 freshly isolated cells and 20 expelled cells were measured in this way. These analyses were conducted on days 1, 2, and 3 at 27°C, day 9 under transitional temperature conditions, and days 11 and 13 at 30°C. The samples were kept in the dark for more than 30 min to relax PSII. One may expect *Fv/Fm* to decrease after the release from coral and exposure to seawater for 2 h. To address this possibility, we monitored any fluctuation of the *Fv/Fm* in freshly isolated *Symbiodinium* and confirmed it to be steady.

### Transmission electron microscopy

To determine the difference between the intracellular structures of normal and degraded expelled *Symbiodinium* cells, transmission electron microscopy was used. Volumes of 5 mL of concentrated samples collected on the first day at 27°C and on the sixth day at 30°C were fixed with 2.5% glutaraldehyde and 0.1 M sucrose in 0.1 M cacodylate buffer (pH 7.4) and kept at 4°C. The fixed *Symbiodinium* were collected by centrifugation and then embedded in agarose gel, followed by post-fixation in 1.5% OsO_4_, dehydration in ethanol series, and resin embedding according to Hikosaka-Katayama et al. (2012) [42]. Ultrathin sections were obtained with an ultramicrotome (ULTRACUT E, Reichert-Jung, Vienna, Austria) using a diamond knife. They were stained with uranyl acetate for 13 min, followed by lead citrate for 4 min, and observed under a transmission electron microscope (JEM-1200EX, JEOL, Tokyo, Japan).

### Measurement of coral surface area using a 3D camera

**Figure 1:**
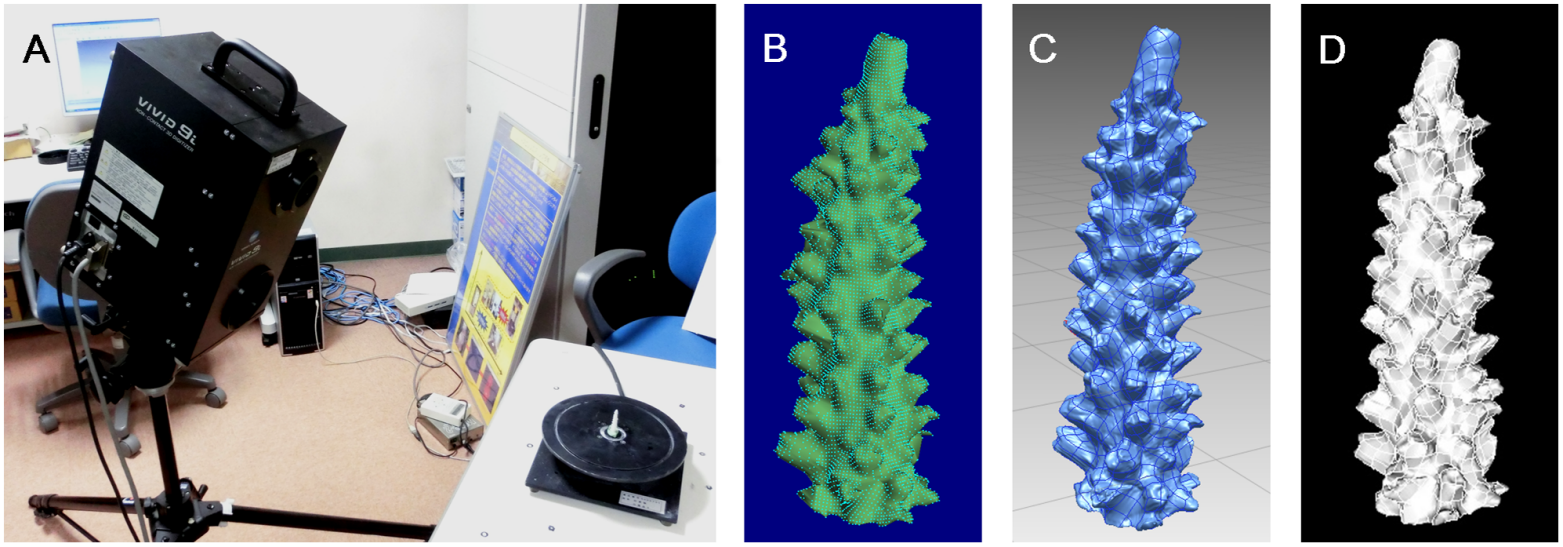
3D camera and 3D images of coral branches of *Acropora muricata*. (A) A 3D camera (VIVID 9i, KONICA MINOLTA) was used for measuring coral surface area. (B) A 3D image from the Polygon Editing Tool software used with polygon and dot data. (C) A 3D image from the rapidform 2006 software used with surface data. (D) A 3D image from the NX I-deas software used for surface area calculation.

To calculate the number of expelled *Symbiodinium* per unit coral surface area, the surface areas of the corals must be measured. Many methods designed to measure coral surface area have been developed so far, such as aluminum foil [43], latex coating [44], paraffin wax coating [45], methylene blue [46], [47], 3D modeling [48]–[51], and computed tomography (CT) [52]. However, some methods are imprecise, while others are not feasible to measure complex branching colony. In this study, we also used a 3D camera to measure surface areas of the coral branches but newly determined the relationship between the surface areas of the pieces and their skeletal weights; in this way, one can theoretically estimate whole colony surfaces simply by weighting the coral. The coral surface areas were measured using a three-dimensional (3D) camera (VIVID 9i, KONICA MINOLTA, Tokyo, Japan) (Figure 1A). Because the entire surface area of a branched coral is impossible to measure even using this system, various small representative skeleton pieces of *Acropora selago* and *Acropora muricata* were individually measured using a 3D camera. Additionally, the same pieces of coral skeleton were weighed, and the relationship between the exact surface area and weight was obtained and used to estimate the total surface area of a whole colony from the weight. In this method, we assumed that the surface area-weight relationships obtained from pointed tips and cylindrical bases of the skeleton might differ. Therefore, more than 10 pieces each of tip and basal sections were separately retrieved from the coral skeletons and measured in 3D. Five photographs from different 60° views were taken and merged into a 360° view using Polygon Editing Tool software (ver 2.10, KONICA MINOLTA, Tokyo, Japan) (Figure 1B). The data showed a composite of dots with XYZ locations. The dot data were then converted to surface data using the rapidform 2006 software (ver. 2006, INUS Technology, Seoul, Korea) (Figure 1C), and the surface area of the coral branches was calculated using the NX I-deas software (ver. 6.2, Siemens PLM software, Plano, TX, USA) (Figure 1D). The relationship between coral surface area and coral skeletal weight was examined, and regression formulae were used. For comparison, another method for coral surface area determination using agar and methylene blue coating [47] was used on the same skeletons.

### Statistical analysis

A one-way repeated measures ANOVA was used to determine whether the numbers of expelled *Symbiodinium* cells differed between the three temperature conditions (non-thermal stress conditions: 27°C, transitional temperature conditions: 27–30°C, and moderate thermal stress conditions: 30°C) and whether *Symbiodinium* densities *in hospite* differed between sampling days. Holm’s method was used to detect differences. A chi-square test (*x* ^2^ test) was performed to identify differences in the percentages of hourly expulsion of *Symbiodinium* relative to the density *in hospite* between sampling days as well as in the proportions of normal and degraded cells in expelled and *in hospite* populations between the three temperatures conditions (27°C, transitional temperature, and 30°C). Additionally, differences in the *Fv/Fm* frequency of freshly isolated and expelled *Symbiodinium* among the different temperature conditions (27°C, transitional temperature, and 30°C) and between the freshly isolated and expelled *Symbiodinium* were examined using a chi-square test (*x* ^2^ test). All tests were performed at the 5% significance level.

## Results

### Coral surface area

**Figure 2:**
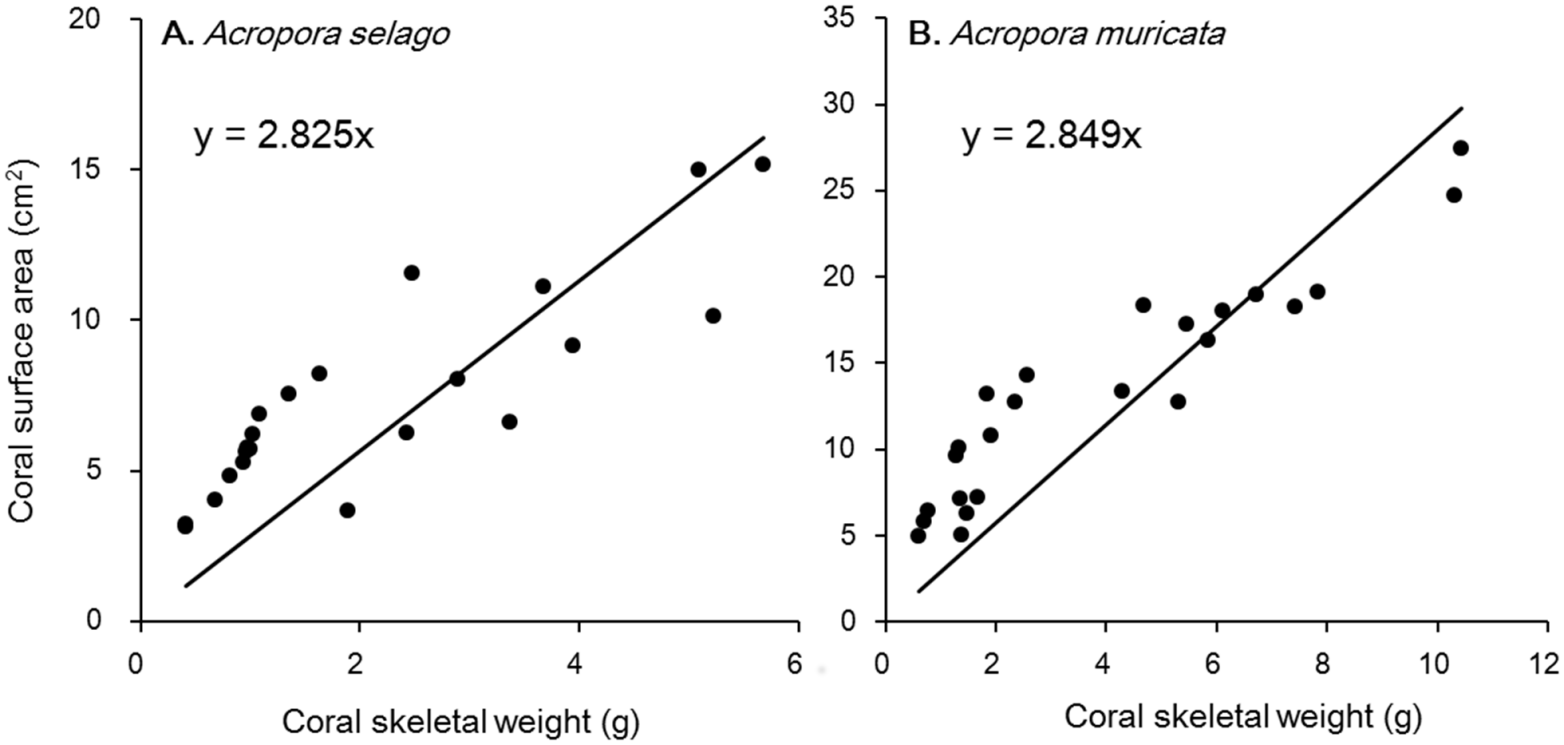
Relationship between coral skeletal weight and surface area. (A) *Acropora selago*. (B) *Acropora muricata.* Regression formulae are shown in each graph.

A positive correlation between the coral surface area and skeletal weight was obtained (*r* = 0.86 in *Acropora selago*, *r* = 0.94 in *Acropora muricata*) (Figure 2). The correlation was high in the analysis combining the data from tip and basal pieces of skeletons, indicating that surface area per unit weight was nearly constant regardless of the region measured. Additionally, a positive correlation was obtained between the 3D camera measurements and the methyl blue coating method (*r* = 0.90 for *Acropora selago*, *r* = 0.95 for *Acropora muricata*). Based on the regression obtained by 3D measurements, the following formulae were developed: y = 2.825x for *Acropora selago* and y = 2.849x for *Acropora muricata*, where y is coral surface area (cm^2^) and x is coral skeletal weight (g). These formulae were used to calculate the coral surface area for entire colonies.

### Number and morphology of expelled and *in hospite Symbiodinium*

In the control colonies maintained at constant 27°C, *Fv/Fm* values of the symbionts were as high as 0.72 (*Acropora selago*) and 0.67 (*Acropora muricata*) even at the end of experimental period (day 13). Thus we concluded the factors other than temperature did not give any negative effect for corals nor symbionts, and the results presented below are showing the effect of temperature.

**Figure 3:**
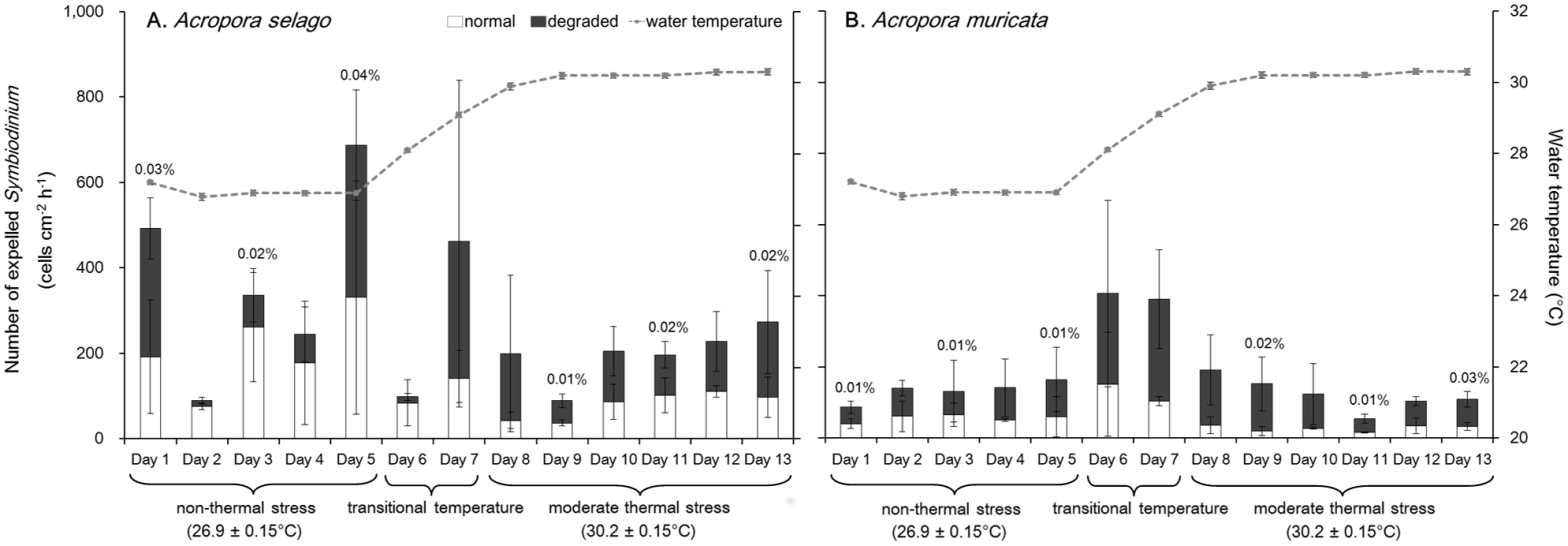
Number of expelled *Symbiodinium* cells from corals. Bars show the numbers of normal (white) and degraded (black) forms of *Symbiodinium*. Dotted lines show water temperature (average of 6 aquaria ± SD). Percentages of expelled cells versus cell density *in hospite* are given above the bars. (A) *Acropora selago*. (B) *Acropora muricata*. Error bars indicate the standard deviations based on triplicate experiments. Three temperature periods with water temperature (mean ± SD among the day) were shown below the graphs.

The numbers of expelled *Symbiodinium* cells from the two coral species are shown in Figure 3. The expulsion rates of *Symbiodinium* at the three temperature conditions, i.e., 27°C (days 1–5), transitional temperature (days 6–7), and 30°C (days 8–13), were not different in either *Acropora selago* or *Acropora muricata* (one-way repeated measures ANOVA: *p*≥0.05 for all comparisons). The average expulsion rates at 27°C were 369±227 (mean ± SD) and 132±29 cells cm^−2 ^h^−1^ in *Acropora selago* and *Acropora muricata*, respectively, and 224±88 and 122±47 cells cm^−2 ^h^−1^ at 30°C, respectively. This indicated, at least under given condition at 30°C, the corals did not exhibit significant increase of the expulsion, which often observed under harsh temperature raise. The percentages of *Symbiodinium* expelled per hour in relation to their density in the coral tissue are shown above the bars in Figure 3 and were constant throughout the experiment. These percentages ranged from 0.01–0.04% and 0.01–0.03% in *Acropora selago* and *Acropora muricata*, respectively, and did not show temporal changes (*x* ^2^ test: *p*≥0.05 for all comparisons).

**Figure 4:**
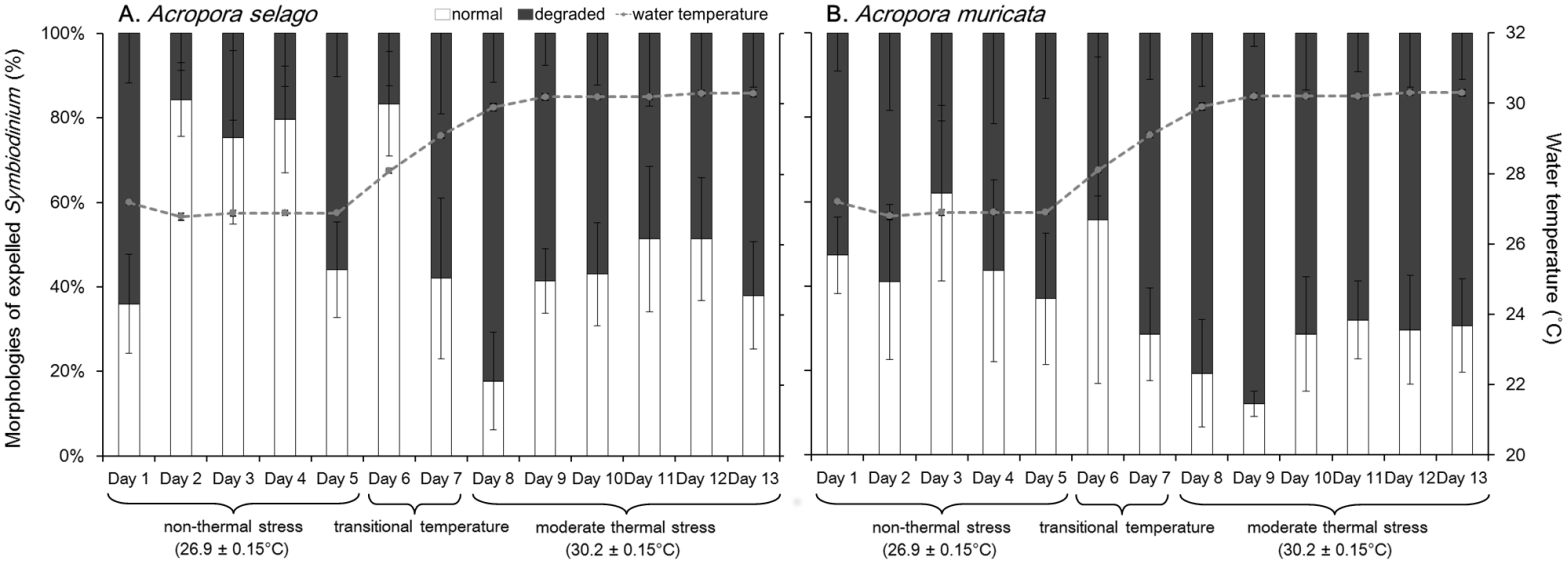
Composition of *Symbiodinium* cells of different morphology expelled from corals. Bars show the percentages of normal (white) and degraded (black) forms of *Symbiodinium*. Dotted lines show water temperature (average of 6 aquaria ± SD). (A) *Acropora selago*. (B) *Acropora muricata*. Error bars indicate the standard deviations based on triplicate experiments. Three temperature periods with water temperature (mean ± SD among the days) were shown below the graphs.

The expulsion rates of normal and degraded cells are shown in Figure 3, while cell composition is summarized in Figure 4. The proportions of both cell types were significantly different between the periods of 27 and 30°C and between those of transitional temperature and 30°C in both coral species (*x* ^2^ test: 27°C and transitional temperature *p* = 0.88, 27°C and 30°C *p* = 6.8×10^−4^, and transitional temperature and 30°C *p* = 0.0011 in *Acropora selago*, *p* = 0.57, *p* = 0.0019, and *p* = 0.011, respectively, in *Acropora muricata*). The mean proportion at 27°C (average of days 1–5) was 64±22% normal cells and 36±22% degraded cells in *Acropora selago* and 46±10% and 54±10% in *Acropora muricata*, respectively. At 30°C, the degraded cells increased to 60±12% and 75±8% in *Acropora selago* and *Acropora muricata*, respectively.

**Figure 5:**
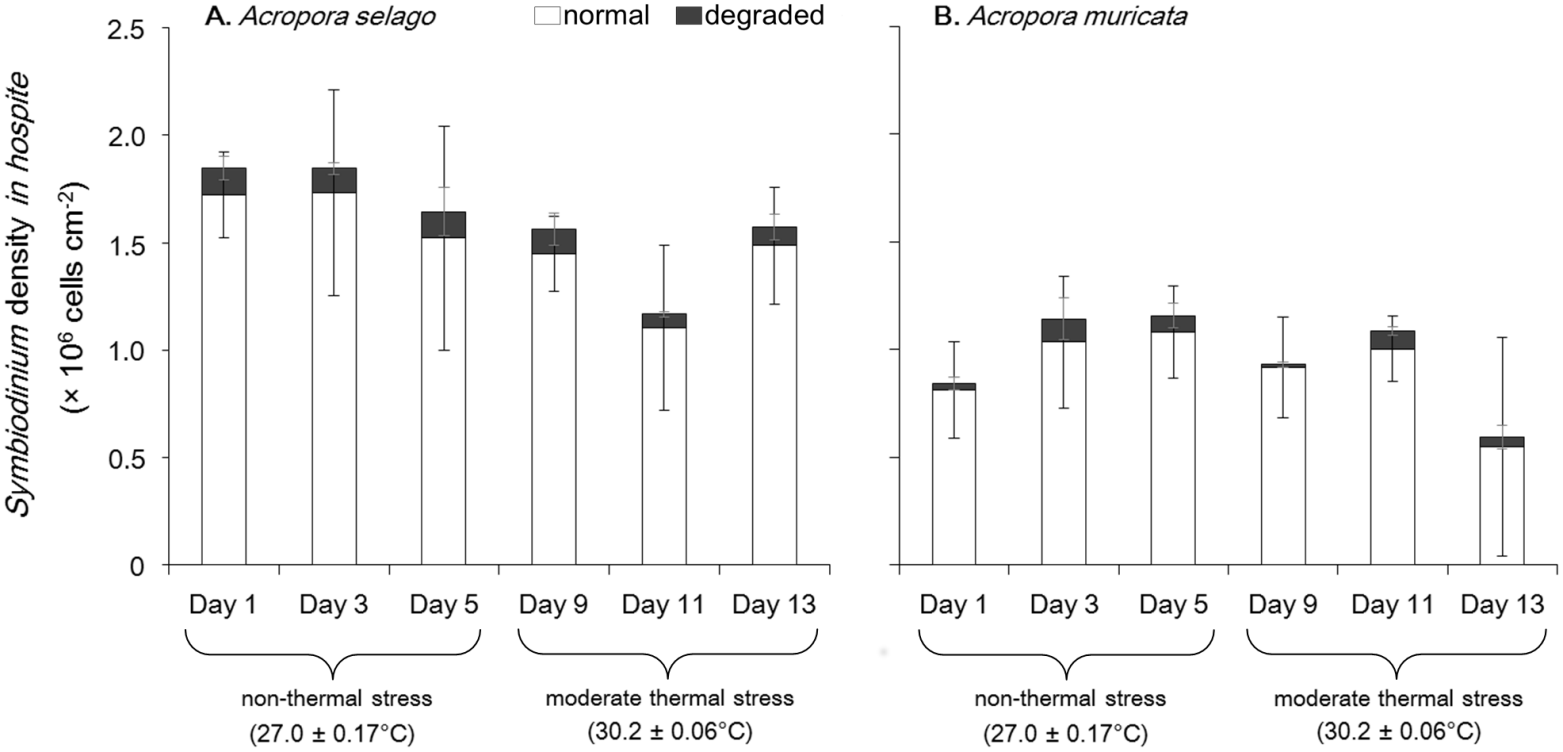
*Symbiodinium* density *in hospite*. Bars show the numbers of normal (white) and degraded (black) forms of *Symbiodinium*. (A) *Acropora selago*. (B) *Acropora muricata*. Error bars indicate the standard deviations based on triplicate experiments. The samples were collected under non-thermal stress period (27.0±0.17°C (mean ± SD among the days); days 1, 3, and 5), and under moderate thermal stress period (30.2±0.06°C; days 9, 11, and 13).

*Symbiodinium* densities *in hospite* (number of cells per coral surface unit) varied from 1.2–1.8×10^6^ cells cm^−2^ in *Acropora selago* and 0.6–1.1×10^6^ cells cm^−2^ in *Acropora muricata* (Figure 5) and did not show any significant differences between sampling days in either species (one-way repeatsupp measures ANOVA: *p*≥0.05 for all comparisons) and corals maintained constant *Symbiodinium* densities during the experiments. Normal cells accounted for 94±1% of the cells in *Acropora selago* and 94±3% of those in *Acropora muricata* (average for all sampling days) and the proportion of cell types did not differ over time in either coral species (*x* ^2^ test: *p*≥0.05 for all comparisons).

### PSII maximum quantum yield (*Fv/Fm*) of freshly isolated and expelled *Symbiodinium*

**Figure 6:**
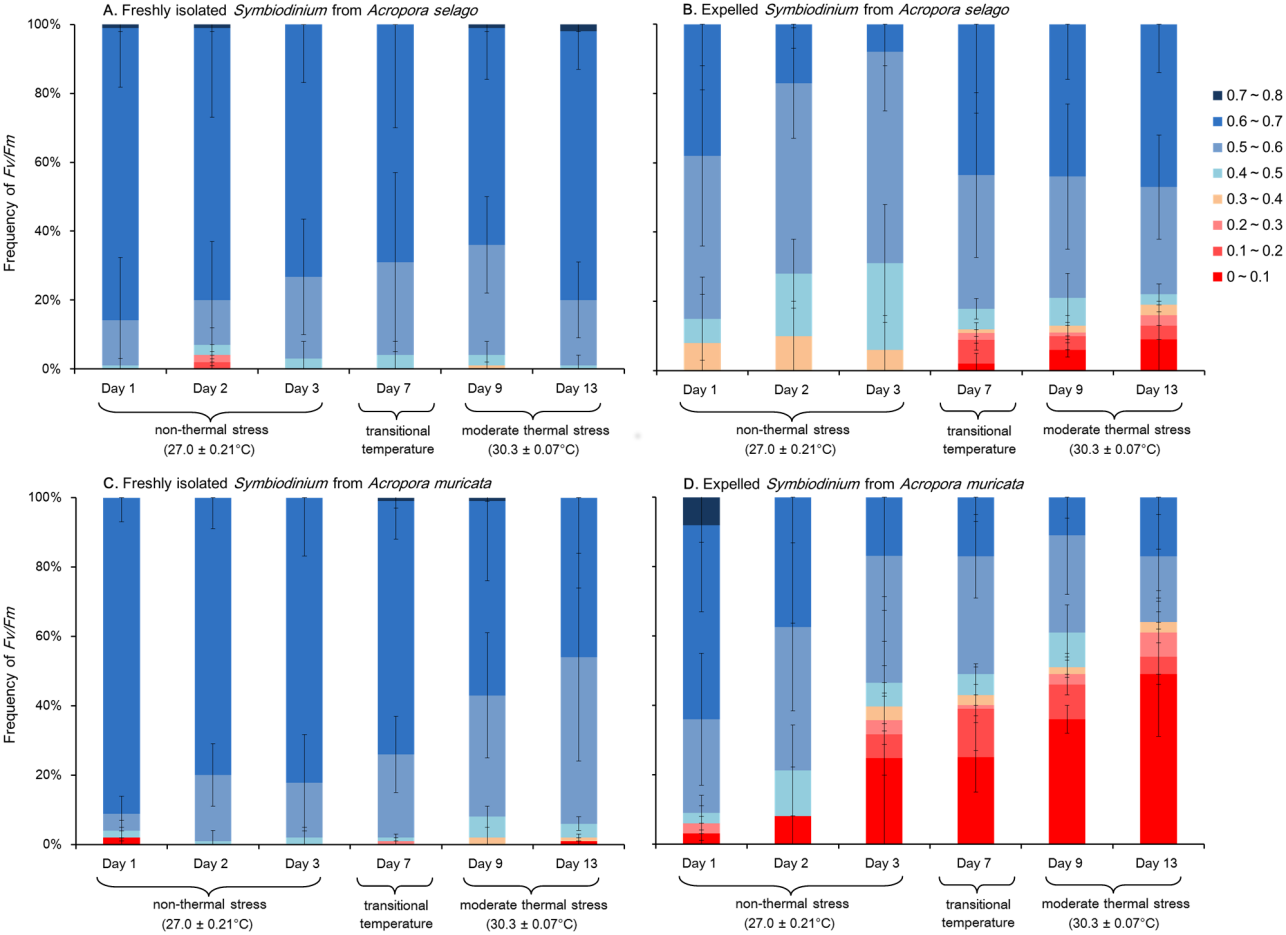
Frequency of *Fv/Fm* for freshly isolated (left panels) and expelled (right panels) *Symbiodinium* from corals. (A) Freshly isolated *Symbiodinium* from *Acropora selago*. (B) Expelled *Symbiodinium* from *Acropora selago*. (C) Freshly isolated *Symbiodinium* from *Acropora muricata*. (D) Expelled *Symbiodinium* from *Acropora muricata*. Error bars indicate the standard deviations based on triplicate experiments.

The frequencies of *Fv/Fm* of the freshly isolated (A and C) and expelled *Symbiodinium* cells from corals (B and D) are shown in Figure 6. Freshly isolated *Symbiodinium* show a large proportion of cells with high *Fv/Fm* values (i.e., ≥0.6). The cells showed higher *Fv/Fm* values than 0.6 in 80±6% of the *Acropora selago* symbionts and 85±6% of the *Acropora muricata* symbionts at 27°C (average of three days). These values were sustained at 30°C: 72±6% (*Acropora selago*) and 52±4% (*Acropora muricata*) (average of two days). The frequencies of *Fv/Fm* were not significantly different between all given temperature conditions for *Acropora selago* (*x* ^2^ test: *p* = 0.42); however, they were slightly but significantly different between 27°C and 30°C and between transitional temperature and 30°C in *Acropora muricata* (*x* ^2^ test: 27°C and transitional temperature *p* = 0.19, 27°C and 30°C *p* = 3.5×10^−5^, transitional temperature and 30°C *p* = 0.017).

The *Fv/Fm* of expelled *Symbiodinium* was always lower than that of freshly isolated *Symbiodinium* at the same temperature conditions in both corals (*x* ^2^ test: *p*<0.05 for all comparisons). Cells showing *Fv/Fm* values of 0.5–0.6 were the main forms expelled from both corals at 27°C (54±7% in *Acropora selago* and 35±7% in *Acropora muricata*, average for three days at 27°C), whereas freshly isolated *Symbiodinium Fv/Fm* values were greater by approximately 0.1 (79±5% in *Acropora selago* and 85±6% in *Acropora muricata*; *Fv/Fm* = 0.6–0.7 in both corals). This trend became obvious with increasing temperature. Although only 8±3% of the expelled cells showed a lower *Fv/Fm* of 0.4 at 27°C (average for three days), this percentage increased to 18±4% by day 13 (sixth day at 30°C) in *Acropora selago* and from 18±19% to 64±4% in *Acropora muricata*. The frequencies of *Fv/Fm* for expelled cells were significantly different between 27°C and transitional temperature and between 27°C and 30°C in both corals (*x* ^2^ test: 27°C and transitional temperature *p* = 1.5×10^−5^, 27°C and 30°C *p* = 2.9×10^−6^, transitional temperature and 30°C *p* = 0.43 in *Acropora selago* and *p* = 5.9×10^−4^, *p* = 1.6×10^−6^, *p* = 0.084, respectively, in *Acropora muricata*).

### Transmission electron microscopy

**Figure 7:**
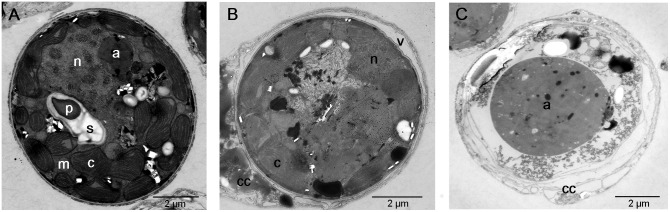
Transmission electron micrographs of expelled *Symbiodinium* from *Acropora selago* on the first day at 27°C. (A) A normal cell with distinct subcellular organelles. (B) A cell undergoing degradation with indistinct subcellular organelles and a thick coral cell. (C) A degraded cell with an enlarged accumulation body and shrunken morphology. Abbreviations: nucleus (n), chloroplast (c), mitochondrion (m), starch (s), pyrenoid (p), accumulation body (a), coral cell (cc), and vacuole (v).

The transmission electron micrographs showed differences in the intracellular structures between a normal cell, a cell undergoing degradation, and a degraded cell obtained on the first day at 27°C (Figure 7). In the normal cell (A), chloroplast thylakoids, starch, and condensed chromosomes were obvious in the nucleus, indicating that the cell was performing active photosynthesis. A thick vacuolated coral cell surrounded the cell undergoing degradation (B), and the subcellular organelles in the degrading cell became indistinct. Finally, the organelles of the degraded cells nearly deteriorated, the cell membrane disappeared, the accumulation body became enlarged, and the cell size shrank to approximately half of the normal size (C).

## Discussion

We found that 1) the corals expelled both degraded and normal *Symbiodinium* cells at both 27°C (non-thermal stress) and 30°C (moderate thermal stress), 2) degraded cells predominated at 30°C, and 3) the proportion of expelled normal cells showing lower *Fv/Fm* values increased at 30°C, whereas cells remaining in the coral tissue were photosynthetically competent even at this temperature.

The expulsion of degraded *Symbiodinium* under non-stressful conditions was also observed in our previous study [32], and we clarified that this phenomenon is a normal and common process for several species of corals for maintaining *Symbiodinium* density *in hospite*. Titlyanov et al. (1996) [53] reported that corals exocytose excess *Symbiodinium* populations from the gastrodermal cells into the body cavity and digest them by phagocytosis at the mesenterial filaments. They also found that the numbers of dividing and degraded cells were similar and concluded that corals regulate *Symbiodinium* density by digesting and expelling the excess population.

Corals expel normal and degraded forms of *Symbiodinium* simultaneously, but the biological significance of this mechanism has not been elucidated. Given that the expulsion of normal forms of *Symbiodinium* exceeded that of degraded forms for several days at 27°C, there must be some inherent purpose to expelling healthy *Symbiodinium* together with degraded cells. Baghdasarian and Muscatine (2000) [54] observed that the dividing cells were preferentially expelled from the corals, the rate of algal expulsion correlated to the rate of algal division, and concluded that expulsion of algae is one of the primary regulators of symbiont population density in host. Stimson and Kinzie (1991) [45] reported that the amount of expelled *Symbiodinium* (presumably cells with normal morphology) increased under light. Additionally, it is known that the release of mucus and mucus lipids from corals was enhanced by light [55] and this seems in order to extrude excess organic matter derived from *Symbiodinium* photosynthesis. Therefore, we speculate that the expulsion of normal forms of *Symbiodinium* may have the same role as that of releasing mucus and mucus lipids, i.e., to extrude excess fixed carbon. This process is obviously different from that of *Symbiodinium* cell digestion, which can increase carbon incorporation. We conclude that the expulsion of both degraded and healthy *Symbiodinium* may be a normal process by which corals maintain *Symbiodinium* density and a constant amount of organic matter within their tissues. Also, *Symbiodinium* density was kept constant during the experiment and those fit to the ranges of densities reported by many other researchers [56]–[60], therefore we believed that corals maintained steady-state level of *Symbiodinium* density during the entire experiment by their normal function for regulating the density.

Previously, we found large numbers of normal cells in the aquarium water at 32°C, which may be attributed to host cell detachment under harsh thermal conditions [32]. The expulsion observed under moderate thermal stress (30°C) may not have occurred due to the same mechanism because we did not observe any cnidocyte release, which is an indicator of host cell detachment [29]. However, at 30°C, the proportion of degraded cells in the expelled populations increased. This result may be explained by the corals’ active digestion and expulsion of damaged *Symbiodinium* under thermal stress. Downs et al. (2009) [31] reported that the digestion of *Symbiodinium* was enhanced by thermal stress. Hill and Ralph (2007) [40] observed the expulsion of *Symbiodinium* under several thermal conditions, and at exposure to 30°C (within the first 24 h), expelled populations were mainly composed of normal cells. The degraded forms among the expelled population increased in number with prolonged exposure to 30°C. We also found that the proportion of degraded cells *in hospite* was low, whereas it was high in expelled populations. These reports, together with our current findings, also support the idea that corals actively digest damaged *Symbiodinium* and selectively expel those degraded cells under moderate thermal stress conditions. Transmission electron microscopy convincingly demonstrated degradation and supported previous findings regarding the digestion process (e.g., [12], [31], [61], [62]). Digestion is marked by a sequence of events such as increased vacuolization between the coral cell and *Symbiodinium*, reduction and condensation of the *Symbiodinium* cell, enlargement of the accumulation body, and disorganization of subcellular organelles.

Apart from the mechanism for degraded cell expulsion, we must consider another mechanism to explain why morphologically normal but photosynthetically incompetent cells increased among the expelled population at 30°C. Corals may employ a rapid process to selectively eliminate damaged cells. This assumption is supported by the sustained high *Fv/Fm* values among *Symbiodinium* residing in the coral tissue during temperature increase, while the values in the expelled population decreased significantly under thermal stress. It has been reported that active forms of oxygen are produced through photosynthesis under thermal stress conditions and that they cause the deterioration of *Symbiodinium* photosystems [63]–[65]. Additionally, Lesser (1997) [27] reported that oxidative stress (production of superoxide radicals and hydrogen peroxides) at elevated seawater temperatures affected both *Symbiodinium* and corals by decreasing photosynthetic performance and enhancing the expulsion of *Symbiodinium* through exocytosis. Therefore, we suggest that corals expel morphologically normal but photosynthetically incompetent cells to immediately extrude these damaged cells without digestion, therefore preventing their accumulation and the resulting oxidative stress. Additionally, Baird et al. (2008) [66] suggested that expelling the symbionts might be a defense mechanism for corals against stress. In this study, expulsion occurred even under non-stress conditions; the *Fv/Fm* of expelled *Symbiodinium* at 27°C was still high, however, slightly but significantly lower than that of cells freshly isolated from tissue. This fact further supports the idea that corals selectively release weaken or damaged *Symbiodinium* cells, most likely in response to reactive oxygen species production from photosynthesis. Although corals possess such adaptive mechanisms, prolonged stressful conditions can be lethal, as seen in many of the coral bleaching events. Indeed, damaged *Symbiodinium* accumulated within coral tissues and resulted in coral bleaching, as observed by Brown et al. (1995) [12]. Production of NO (nitric oxide) by hosts also relates to temperature-induced coral bleaching and it is suggested to be up-regulated by oxidative stress in the algae [67]–[70].

In conclusion, we suggest that expulsion mechanisms differed depending on temperature conditions. At 27°C (non-thermal stress conditions), the expulsion of *Symbiodinium* was part of a regulatory mechanism to keep constant *Symbiodinium* density and maintain a stable carbon concentration with expelling either digested and normal form of *Symbiodinium*; this normal process maintains symbiosis. However, at 30°C (moderate thermal stress), *Symbiodinium* become damaged, and corals selectively digest the damaged cells or immediately expel them without digestion by exocytosis, which is most likely an adaptive mechanism in response to moderate thermal stress. Nonetheless, corals are known to show bleaching with prolonged moderate thermal stress. Therefore, if stressful conditions prevail, damaged *Symbiodinium* may accumulate within coral tissues, resulting in coral bleaching.
